# Structure Elucidation of Coxsackievirus A16 in Complex with GPP3 Informs a Systematic Review of Highly Potent Capsid Binders to Enteroviruses

**DOI:** 10.1371/journal.ppat.1005165

**Published:** 2015-10-20

**Authors:** Luigi De Colibus, Xiangxi Wang, Aloys Tijsma, Johan Neyts, John A. B. Spyrou, Jingshan Ren, Jonathan M. Grimes, Gerhard Puerstinger, Pieter Leyssen, Elizabeth E. Fry, Zihe Rao, David I. Stuart

**Affiliations:** 1 Division of Structural Biology, University of Oxford, Oxford, United Kingdom; 2 National Laboratory of Macromolecules, Institute of Biophysics, Chinese Academy of Science, Beijing, China; 3 Laboratory of Virology and Chemotherapy, Rega Institute for Medical Research, Leuven, Belgium; 4 Diamond Light Source, Didcot, United Kingdom; 5 Department of Pharmaceutical Chemistry, University of Innsbruck, Innsbruck, Austria; 6 Laboratory of Structural Biology, School of Medicine, Tsinghua University, Beijing, China; Purdue University, UNITED STATES

## Abstract

The replication of enterovirus 71 (EV71) and coxsackievirus A16 (CVA16), which are the major cause of hand, foot and mouth disease (HFMD) in children, can be inhibited by the capsid binder GPP3. Here, we present the crystal structure of CVA16 in complex with GPP3, which clarifies the role of the key residues involved in interactions with the inhibitor. Based on this model, *in silico* docking was performed to investigate the interactions with the two next-generation capsid binders NLD and ALD, which we show to be potent inhibitors of a panel of enteroviruses with potentially interesting pharmacological properties. A meta-analysis was performed using the available structural information to obtain a deeper insight into those structural features required for capsid binders to interact effectively and also those that confer broad-spectrum anti-enterovirus activity.

## Author Summary

Hand, foot and mouth disease (HFMD), a common infectious disease of children documented worldwide is caused by enterovirus infection. Coxsackievirus A16 (CVA16) and enterovirus 71 (EV71) are the major causative agents of HFMD. Although EV71 vaccines have completed phase III clinical trials, there remains no vaccine against CVA16 or licensed drug against either CVA16 or EV71. In this study we show the crystal structure of CVA16 in complex with inhibitor GPP3, shedding light on the residues involved in the interaction with the virus capsid. Taking advantage of the crystal structure we employed a quantum mechanics polarized ligand docking (QMPLD) protocol to dock two recently designed capsid binders. These molecules have been already proven to be very powerful inhibitors of EV71 and here we show that they exhibit strong antiviral activity against a wide range of enterovirus.

Our research provides new insights into the design of inhibitors for the treatment of different pathogenic enterovirus infections.

## Introduction

HFMD is caused by enterovirus infections, predominantly CVA16 and EV71[1,2]. This childhood infection is usually mild, but occasionally leads to neurological disease and even death in the most extreme cases. Major outbreaks have been reported in the past, predominantly in Asia, leading to these viruses becoming a growing public health concern. Currently, there is no vaccine or effective drug available for the treatment of these infections[3].

**Fig 1:**
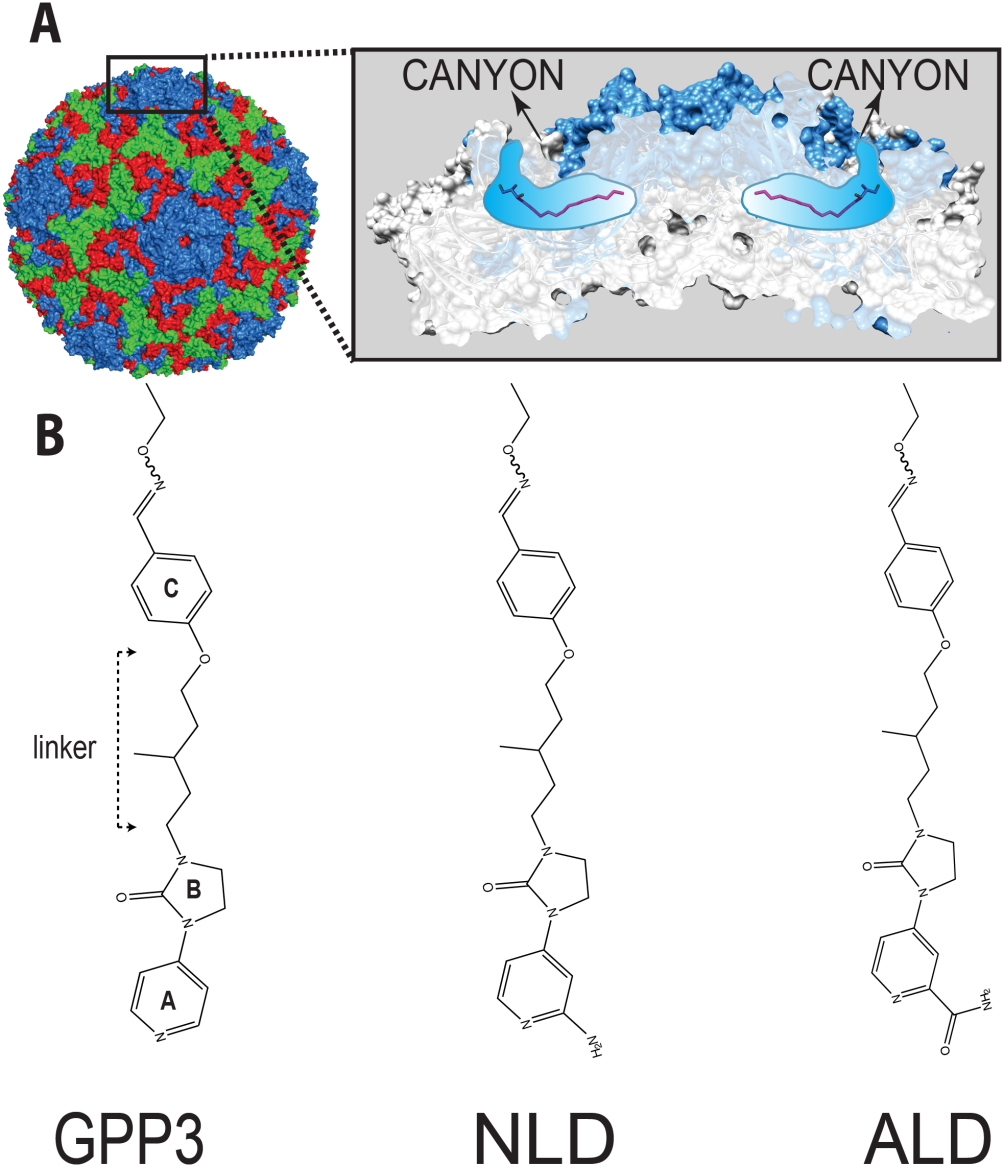
**(A)** CVA16 particle with capsid protein subunits VP1 (blue), VP2 (green), VP3 (red), VP4 (yellow) in surface representation. Inset in (**A**) shows the location of CVA16 inhibitor binding in the pocket (shown schematically in blue) lying below the canyon floor, here occupied by a natural pocket factor (magenta, in sticks representation). The VP1 subunits at the icosahedral five-fold axis are shown as a blue surface overlaid on a cartoon representation whereas the other subunits are in light gray. A segment around the five-fold axis is cut away to reveal two pockets. **(B)** A selection of 3-(4-pyridyl)-2-imidazolidinone derivative structures. The following chemical moieties are labeled in GPP3: A, pyridine ring; B, imidazole moiety; C, phenoxy group.

Enteroviruses belong to the *Picornaviridae* family of small viruses with a single-stranded, positive-sense genomic RNA. The viral genome is enclosed in a non-enveloped icosahedral capsid that is built out of 60 copies of the structural proteins VP1 to VP4. VP1 surrounds the 5-fold axes and VP2 and VP3 alternate around the 2- and 3-fold axes, while VP4 forms part of the inner lining of the capsid. Canyon-like depressions encircle the 5-fold axes and are frequently the sites for receptor attachment[4] (Fig 1a).

Uncoating, the process during which the capsid opens up to release the viral genome into the host-cell cytosol to initiate virus replication, is key to enterovirus infection. Structural analysis has revealed that each of the 60 VP1 proteins in the enterovirus capsid contain a hydrophobic ‘pocket factor’. This is a natural lipid (for instance sphingosine), which is buried in a hydrophobic pocket at the base of the canyon, within the VP1 capsid protein (Fig 1a). Expulsion of this molecule during binding of the virus to its receptor prepares the particle for a cascade of structural rearrangements to open up and release its genome[5–7]. Because expulsion of the pocket factor is required for infection, a molecule that replaces this factor with higher affinity can serve as an antiviral agent that acts before the virus can replicate.

Here, we present the crystal structure of CVA16 in complex with the capsid binder 3-(4-pyridyl)-2-imidazolidinone (GPP3) (Fig 1b), and calculate the energy of the compound/protein interaction using an *in silico* docking method. The same *in silico* protocol is used to dock two recently designed capsid binders[8] into the CVA16 crystal structure to demonstrate that they have a similar binding mode. Furthermore, the structural and *in silico* results are analyzed in the context of the antiviral activity of these inhibitors against a wide range of enteroviruses, showing the potency and broad-spectrum antiviral activity of these molecules.

## Results

### Structural basis of CVA16-GPP3 interactions

**Fig 2:**
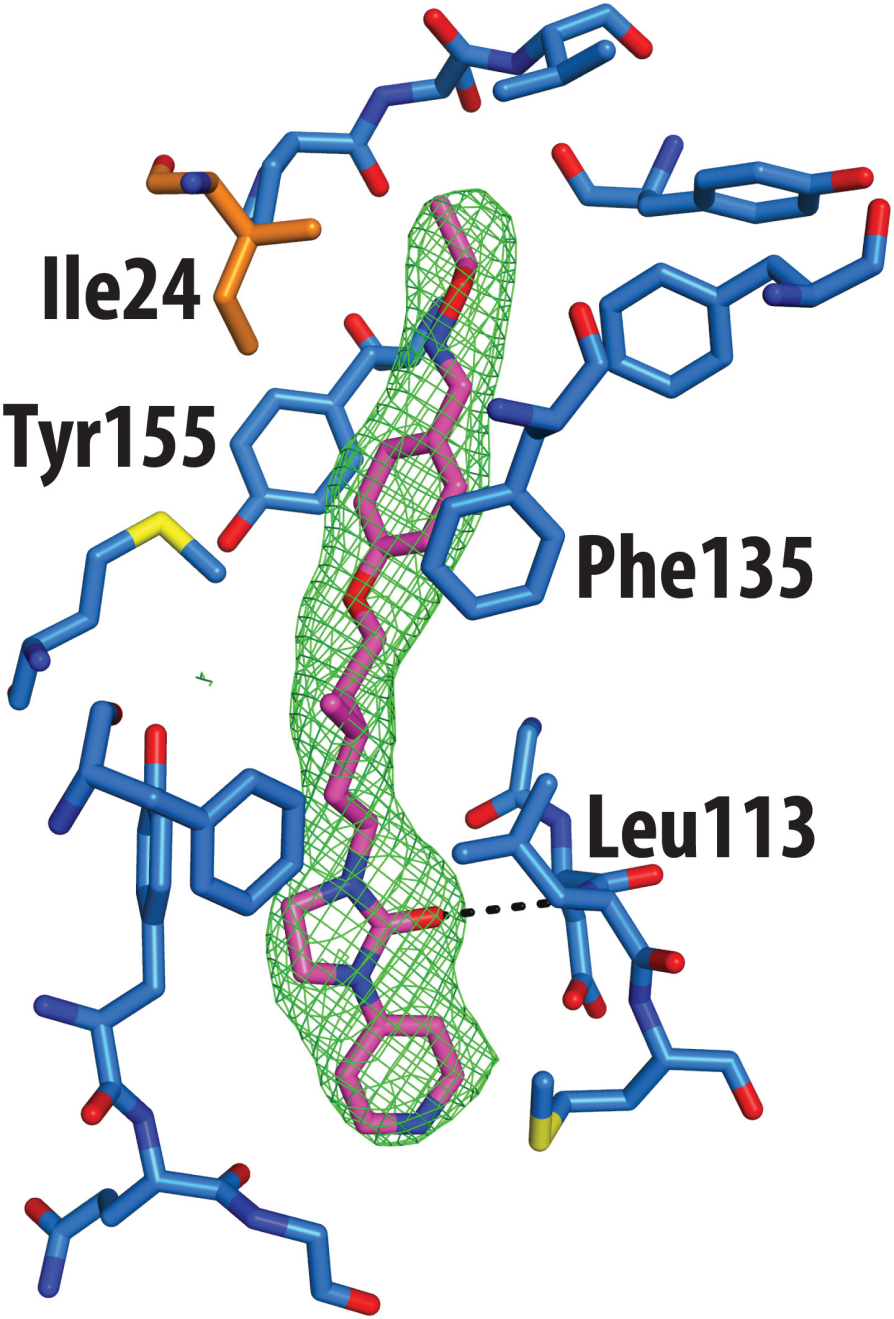
Real-space averaged OMIT |*F*_o_| − |*F*_c_| map (green mesh) of GPP3 inhibitor bound to CVA16. VP1 residues within 3 Å of the ligand are colored in blue and shown as sticks; the side chain of Ile24 of VP3 is colored in orange.

**Fig 3:**
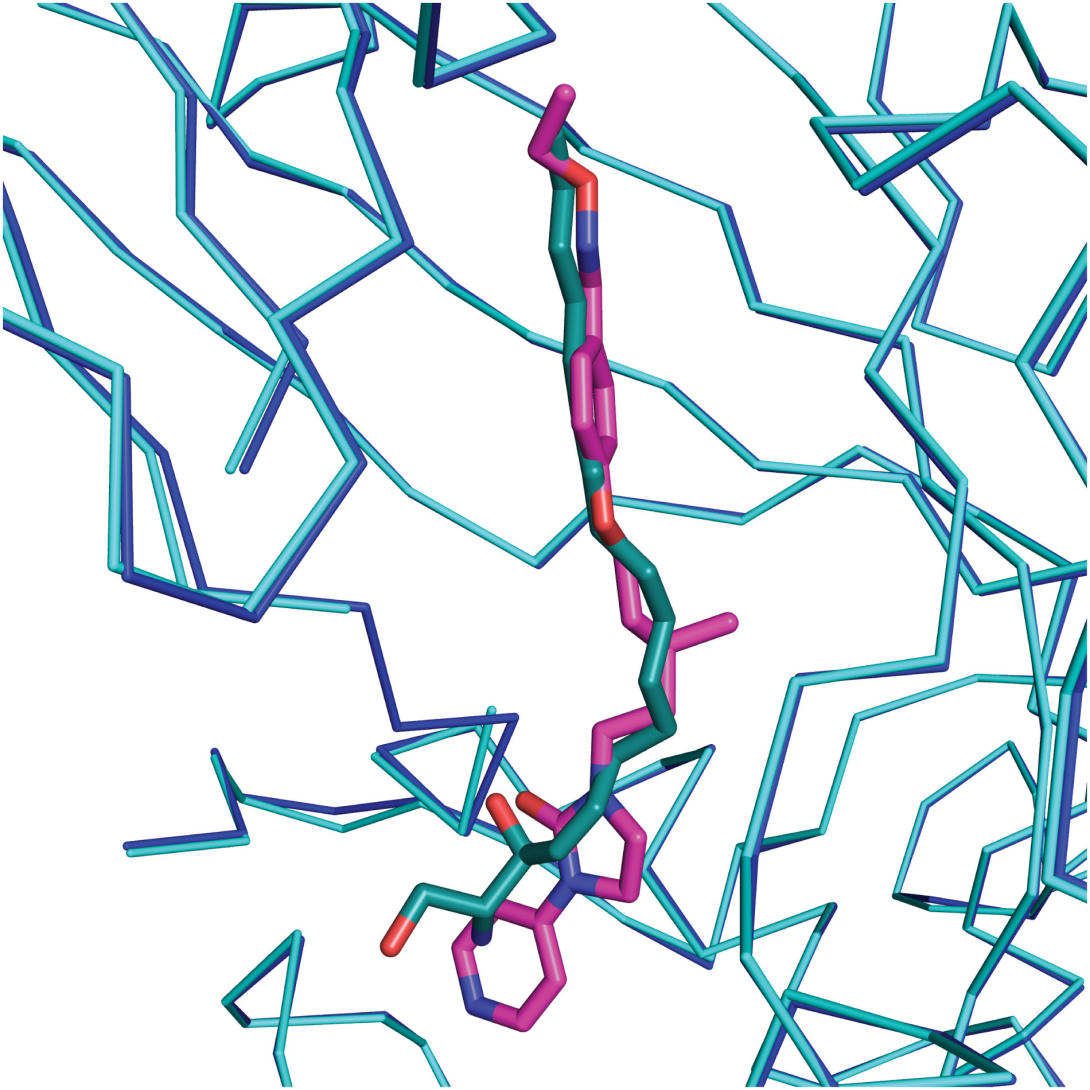
Comparison of complexes of CVA16 with sphingosine and GPP3. Sphingosine (green)–CV16 VP1 (cyan), superimposed on GPP3 (magenta)–CVA16 VP1 (blue). The RMS difference between all Cα atoms of the icosahedral protomer is 0.2 Å.

The crystal structure of CVA16 in complex with the uncoating inhibitor GPP3 was determined by crystallography (Materials and Methods, Fig 2 and Table 1). To this end, CVA16 crystals were soaked with the inhibitor dissolved in DMSO because GPP3, like most pocket-factor analogs, is rather insoluble in water. Diffraction data were collected from 15 crystals at room temperature in crystallization plates at the Diamond Light Source[9]. Data were scaled together, merged and the structure solved at 2.75 Å resolution by molecular replacement using the structure of the mature CVA16 virus[10]. The electron density map revealed that the compound replaces the natural pocket factor (modeled as sphingosine) with only a negligible shift (~ 0.2 Å) in the polypeptide backbone of the residues lining the pocket, reflecting its shape similarity with sphingosine (Fig 3). The surface area accessible to solvent, calculated by Areaimol[11], is 12.3 Å^2^ for GPP3 in the VP1 pocket whereas for the sphingosine it is 11.8 Å^2^. This demonstrates that GPP3 is buried within the pocket. As expected, GPP3 binds with its pyridine ring close to the entrance of the pocket, with the carbonyl oxygen of the imidazole moiety hydrogen-bonding to the backbone nitrogen of residue Ile113, which is also the case for sphingosine, and with the phenoxy ring sandwiched between two hydrophobic residues (Phe135 and Tyr155) (Fig 2). This binding mode was also observed for EV71 in complex with the same compound[8], except that EV71 has a phenylalanine at position 155. The RMSD between EV71 crystal structure and CVA16 structure is 0.5Å for all aligned residues. Their sequence identity is ~80% in the capsid proteins.

**Table 1:** Data collection and refinement statistics.

CVA-16-GPP3	
**Data collection**	
No. crystals (positions)	15(20)
Space group	P4_1_2_1_2
a,b,c (Å)	491.6, 491.6, 709.6
Resolution (Å)^a^	50.0–2.75 (2.85–2.75)
Rpim	0.481
<I/σ(I)>	1.42 (0.621)
Completeness	40.5 (36.1)
Redundancy	1.3 (1.3)
**Refinement**	
Resolution	50.0–2.75
No. reflections	879111/8919
R_work_/R_free_ ^b^	0.305/0.307
No. atoms	
Protein	6398
Ligand/ion/water	42
Average B-factors (Å^2^)	
protein	24
Ligand/ion/water	27
r.m.s. deviations	
Bond lengths (Å)	0.007
Bond angles (°)	1.4
Ramachandran plot outliers (%)^c^	0.25
Ramachandran plot most favored regions (%) ^c^	93.35
Rotamer outliers (%)^c^	1.87

^a^Values in parentheses are for highest-resolution shell.

^b^The *R*_free_ is of limited significance, owing to the considerable noncrystallographic symmetry.

^c^ According to the criterion of Molprobity

### *In silico* docking

**Fig 4:**
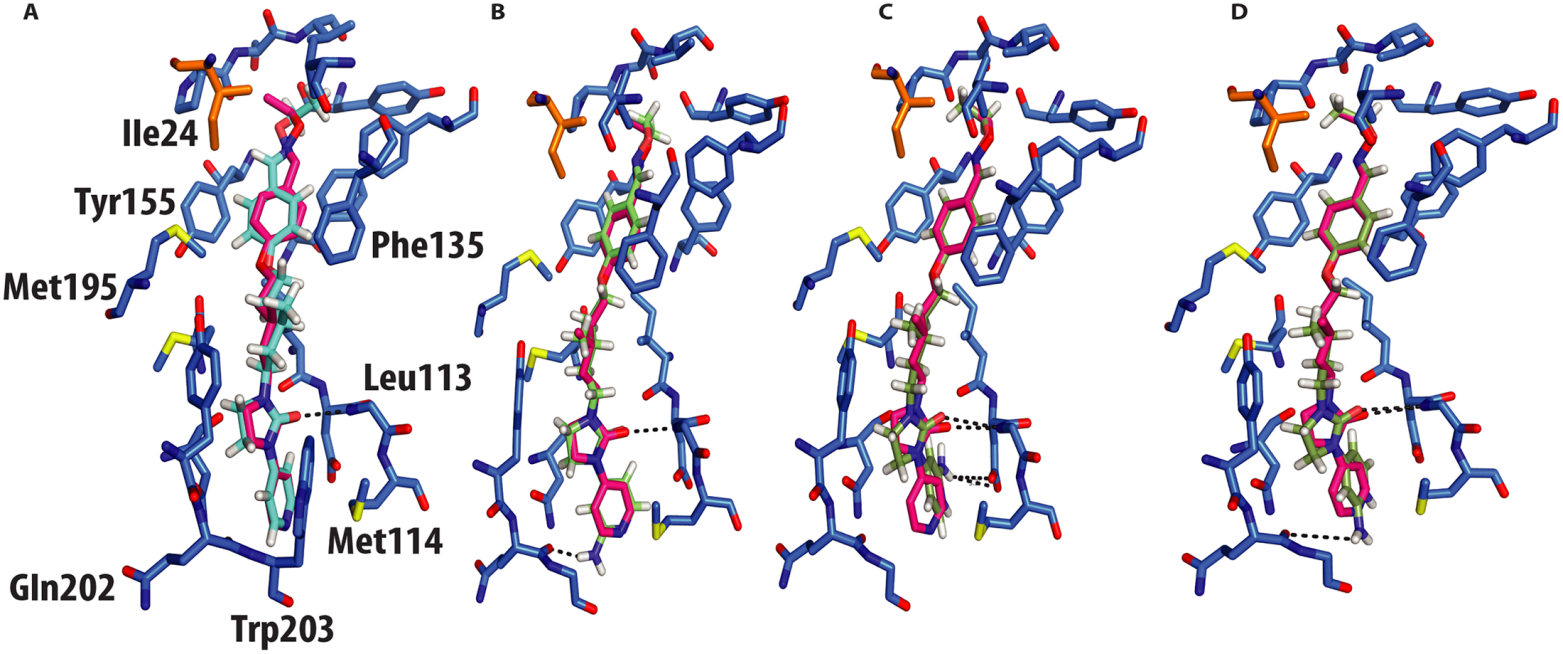
Molecular docking of GPP3 and NLD into the VP1 pocket. Both ligands are shown as sticks. (**A**) Overlay of the docked ligand GPP3 (cyan) by QMPLD and the experimentally determined conformation of GPP3 (magenta) (RMSD between the experimentally observed pose and docked pose for all inhibitor atoms 0.5Å) (**B**) NLD hydrogen-bonds with main chain nitrogen of Gln202 (RMSD between the experimentally observed pose and docked pose for all inhibitor atoms 0.6Å). (**C**) Highest score docking pose of protonated form of NLD hydrogen-bonds with main chain nitrogen of Gln202 (RMSD between the experimentally observed pose and docked pose for all inhibitor atoms 0.4Å). (**D**) Second highest docking score of NLD, note hydrogen bonds with the side chain of Asp112.

*In silico* docking into the CVA16 structure[10] used quantum mechanics–polarized ligand docking (QMPLD)[12] implemented in the Schrödinger suite (http://www.schrodinger.com/) (Fig 4), using procedures described previously[8] (Materials and Methods). The docking of GPP3 in the crystal structure reproduces exactly the experimentally observed pose (Fig 4a) with an energy of interaction of -66 Kcal/mol, expressed as a sum of van der Waals and electrostatic energies. More powerful capsid binders, called NLD and ALD, have been designed against EV71[8]. NLD has an IC_50_ value that is an order of magnitude lower than GPP3, which was previously the best capsid binder reported against this virus. These molecules were docked using the same protocol and the energy of binding calculated.

NLD, docked in the CVA16 pocket, hydrogen-bonded with the main chain oxygen of Gln202 (Fig 4b), with an energy of binding of -69 Kcal/mol, this docking pose was also observed when NLD was docked in the EV71 pocket. This difference in energy is in agreement with the observed difference in EC_50_ values between GPP3 (EC_50_ = 0.014μM) and NLD (EC_50_ = 0.00012μM) (Table 2), the latter having an EC_50_ value two order of magnitude smaller. At pH = 7.0 the pyridine moiety of NLD may also be protonated; so we also docked this molecule into the VP1 pocket. The result was a rotated pyridine still involved in the interaction with the main chain oxygen of Gln202, with a binding energy of -64 Kcal/mol. The second highest scoring docking pose for protonated NLD in CVA16 has the same orientation as that observed in the EV71-NLD crystal structure[8], hydrogen bonding with Asp112 (Fig 4c). Moreover the energy difference between the first and the second docking pose is only 0.29 Kcal/mol. The reason for this discrepancy could be the presence of the Met114 in the CVA16 sequence (Thr114 in EV71), which may hinder the full rotation of the protonated pyridine moiety to hydrogen bond with Asp112 (Fig 4d).

**Table 2:** Antiviral activity of the capsid binders NLD, ALD and GPP3 in virus-cell-based assays against a panel of enteroviruses.

	NLD	ALD	GPP3
***cells***	*CC*_*50*_ *(μM)*		
**BGM**	17 ± 2	7 ± 1	7 ± 2
**HeLa H1**	>22	>21	>24
**HeLa Rh**	5.0 ± 1.1	4.1 ± 0.1	4.1 ± 2.4
**RD**	20 ± 1	33 ± 6	9 ± 3
***Virus***	*EC*_*50*_ *(μM)*		
**CVA9** ^a^	6.0 ± 1.0	5.4 ± 1.0	1.5 ± 0.1
**CVA16** ^b^	0.00012 ± 0.00004*	0.043 ± 0.008*	0.014 ± 0.004*
**CVA21** ^a^	0.013 ± 0.008*	0.056 ± 0.04*	0.016 ± 0.015*
**CVB3** ^a^	2.6 ± 0.2	2.2 ± 0.2	3.7 ± 0.2
**CVB4** ^a^	>17	>7	>7
**PV1** ^a^	0.9 ± 0.4*	3.5 ± 1.4*	0.2
**PV2** ^a^	0.040	0.075 ± 0.019*	0.006
**PV3** ^a^	0.095 ± 0.029*	0.184 ± 0.008*	0.066
**ECHO11** ^a^	1.3 ± 0.2	2.1 ± 0.5	0.5 ± 0.1
**HRV02** ^c^	2.4	>21	1.0 ± 0.7
**HRV14** ^c^	>5.0	0.843 ± 0.013	1.5 ± 0.3
**EV71-B2** ^d^	0.00053 ± 0.00005*	0.031 ± 0.012*	0.00096 ± 0.0006*
**EV71-B5** ^d^	0.0014 ± 0.0003*	0.082 ± 0.023*	0.00093 ± 0.0003*

CVB3 (strain Nancy); CVB4 (strain E2 Edwards); CVA9 (strain Bozek); CVA16 (strain G-10); CVA21 (strain Coe); PV1 (poliovirus type 1 strain Sabin); PV2 (poliovirus type 2 strain Sabin); PV3 (poliovirus type 3 strain Sabin); ECHO11 (echovirus 11 strain Gregory); HRV2 (human rhinovirus type 2); HRV14 (human rhinovirus type 14); EV71-B2 (strain 11316 genogroup B2); EV71- B5 (strain TW/96016/08 genogroup B5).

^a^Assay performed on BGM cells;

^b^Assay performed on HeLa H1 cells;

^c^Assay performed on HeLa H1 cells;

^d^Assay performed on RD cells.

* indicates that at least one treated, infected condition reproducibly resembled the untreated, uninfected cell controls.

### Virus-cell-based assays and structure informed meta-analysis

**Fig 5:**
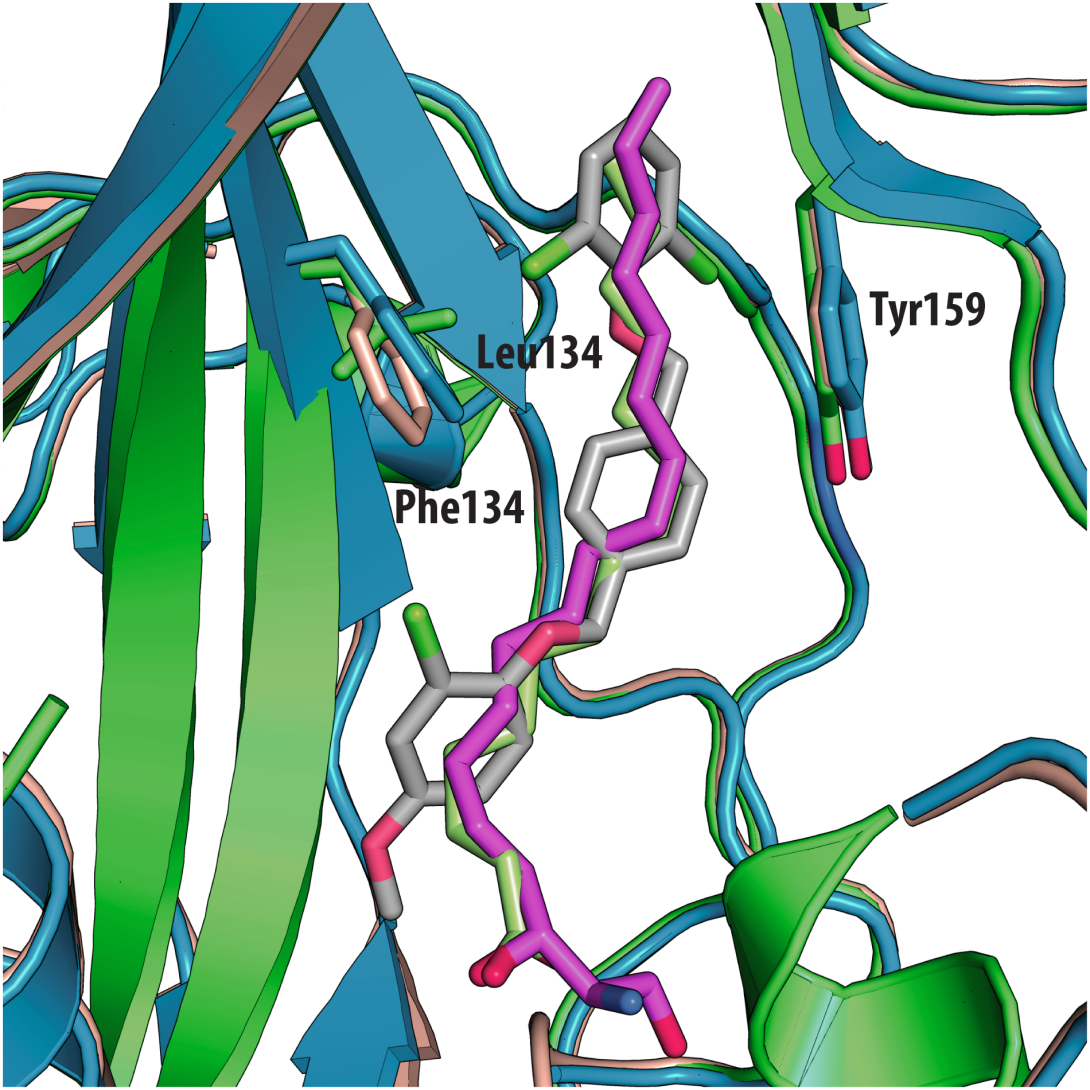
Superposition of VP1 structures of the 3 types of poliovirus (PDBid: 1HXS in green, 1EAH in light orange, 1PVC in blue). All the ligands and side chains in the pocket are shown as sticks. Sphingosine is shown in pink, palmitate in green, inhibitor SCH48973 in gray. The residues shown are Tyr159, Phe134 and Leu134.

The antiviral activity of the three compounds was assessed in virus-cell-based assays against a panel of representative viruses (Table 2). To investigate the relationship between the structure of the VP1 pocket and the different EC_50_ values obtained, a meta-analysis was performed based on the available crystal structures of the viruses[8,13–15] included in the test panel. Perhaps surprisingly, the measured inhibitory activities varied widely across the three types of poliovirus (PV1, PV2, PV3), furthermore GPP3 was a somewhat better inhibitor, for all three types, than either NLD or ALD. The VP1 pocket of the different poliovirus types is shown on Fig 5. The energy of interaction between the pocket factor, identified as a natural lipid, and the residues lining the binding pocket is the combination of hydrophobic and electrostatic terms. In all structures, the lipid, modeled on the basis of the electron density as a sphingosine or palmitate, sits with its hydrophobic tail between two hydrophobic residues (Phe134 and Tyr159 in PV2 and PV3, Leu134 in PV1) at the bottom of the pocket and establishes a hydrogen bond between the polar head of the lipid and the protein main chain of Ser206 and side chain of Tyr112 in PV3 or the side chain of Tyr112 in PV1 at the pocket entrance. In PV1, position 134 is occupied by a leucine residue, while, for PV2 and PV3, a phenylalanine is present. The difference at this position may play a crucial role in the affinity of the interaction with capsid binders because Phe134, together with Tyr159 sandwich the phenoxy moiety present in all the capsid binders in a ‘hydrophobic trap’[8]. Moreover, all three types of poliovirus have a less polar entrance to the pocket, which is less exposed to the solvent compared to that of EV71 and CVA16. This may explain why GPP3, which doesn’t carry any extra polar group on the pyridine moiety, can fit properly into the pocket and is the most potent inhibitor. Nevertheless good antiviral activity was observed for NLD against PV2 and PV3. In these viruses, residues Tyr159 and Phe134, the same as found in CVA16, are involved in the interaction with the phenoxy moiety of NLD while the side chain of Lys113 at the bottom of the pocket can easily adopt a different rotamer conformation. As such, the protein is able to better accommodate the inhibitor and also to establish a hydrogen bond with the amine or amide group on NLD or ALD. Similarly, Thr111 at the bottom of the pocket can hydrogen-bond with the amine or amide group of NLD and ALD, whilst an additional hydrogen bond can be established between Tyr112 and the amine or amide group of NLD and ALD because the pyridine moiety is free to rotate around the bond with the imidazolidinone.

**Fig 6:**
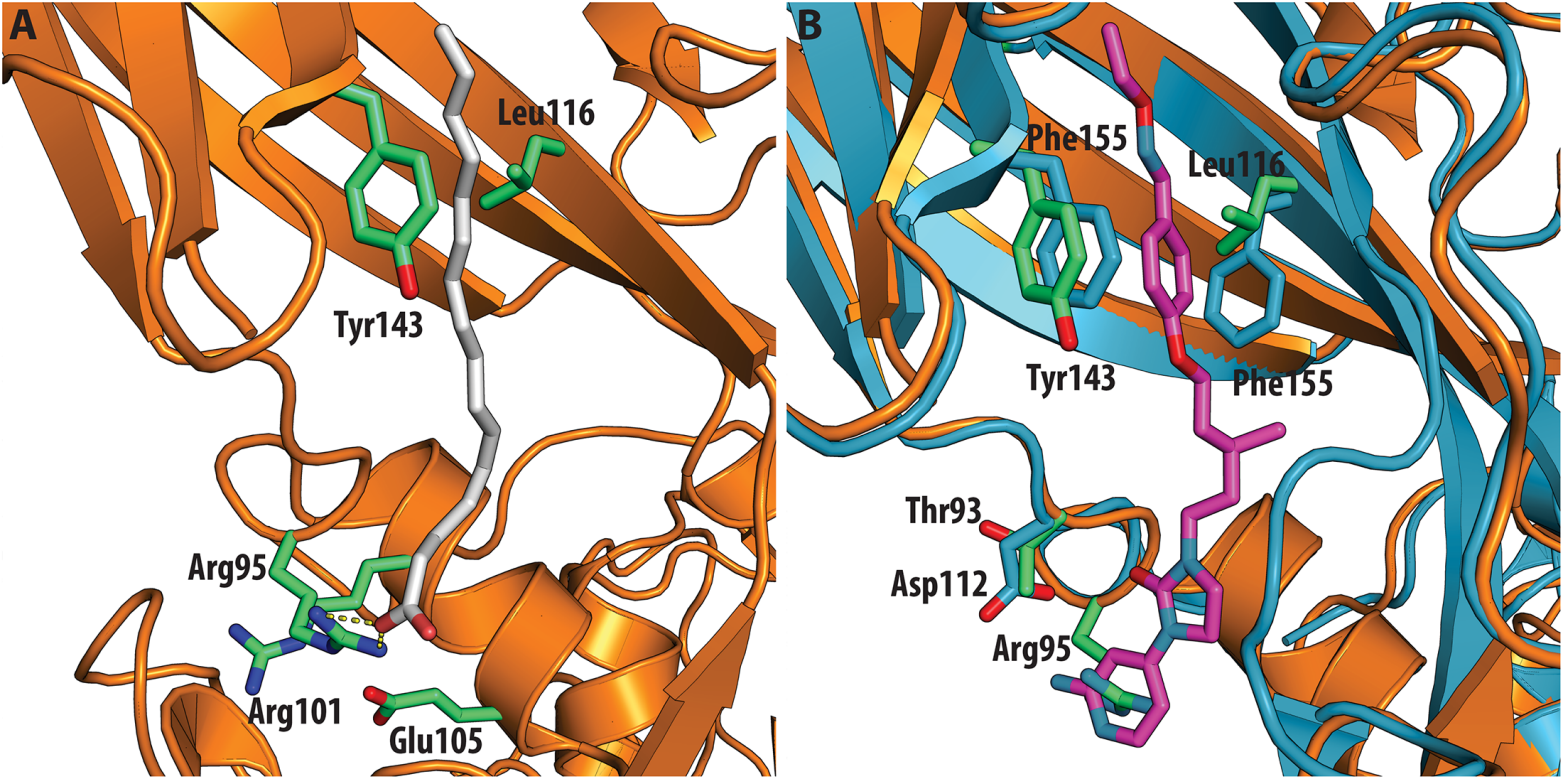
(**A**) VP1 pocket of CVB3. Ligand and residues in the pocket are shown as sticks (PDBid:1COV). (**B**) Superposition of CVB3 (orange) on EV71 (blue) in complex with NLD (PDBid:4CEY), ligand and side chain residues are shown in sticks. Residues shown in EV71 are: Asp112, Thr114, Phe135, Phe155. Residues shown in CVB3 are: Thr93, Arg95, Leu116 and Tyr143.

**Fig 7:**
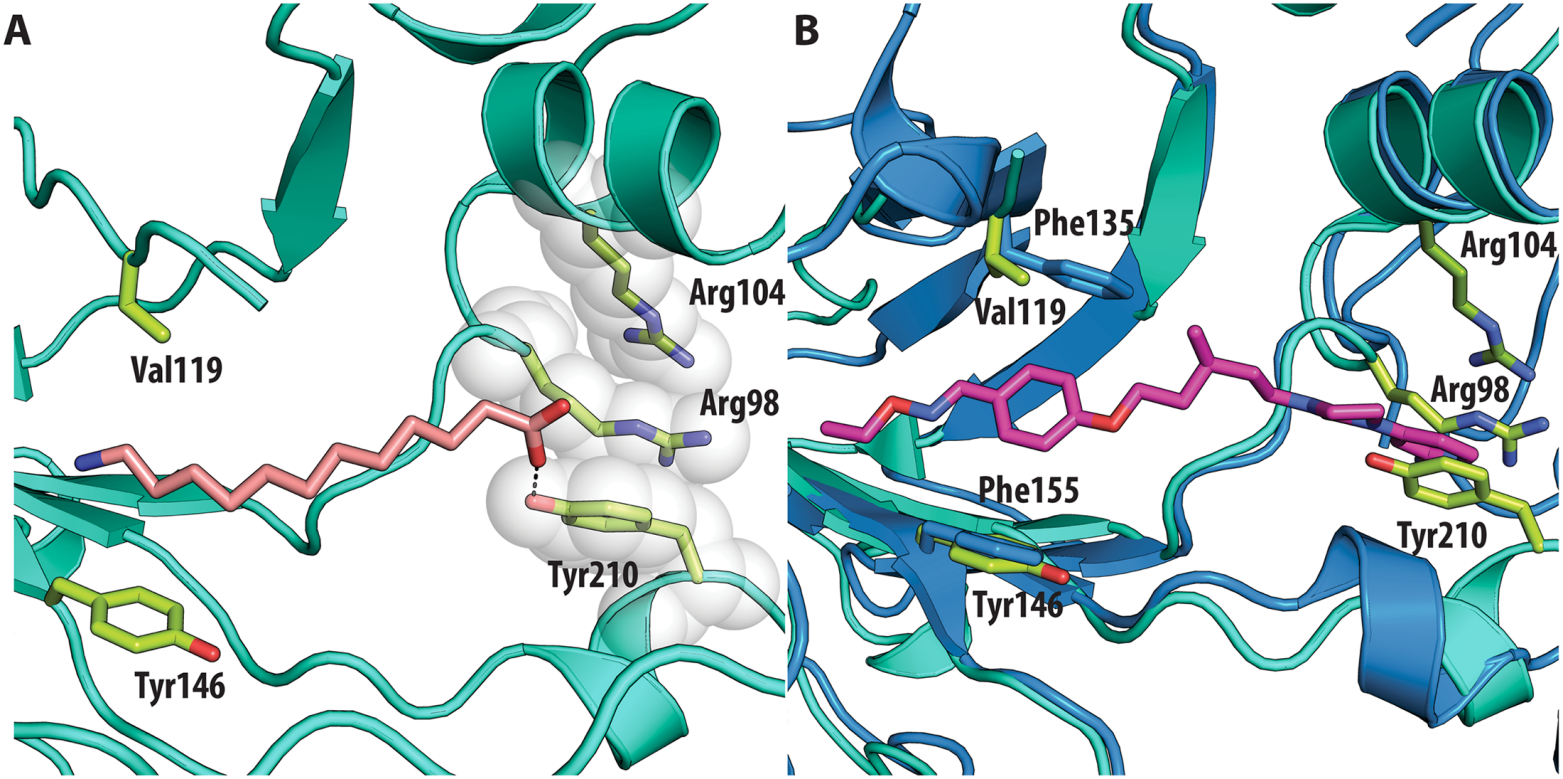
(**A**) VP1 pocket of echovirus11 (EV11). Ligand and residues side chains are shown in sticks (PDBid:1H8T). (**B**) Superposition of EV11 (cyan) on EV71 (blue) in complex with NLD (PDBid:4CEY) ligand and side chain residues are shown in sticks. Residues shown in EV71 are: Asp112, Thr114, Phe135, Phe155. Residues shown in EV11 are: Ser96, Arg98, Arg104, Val119, Tyr146 and Tyr210.

**Fig 8:**
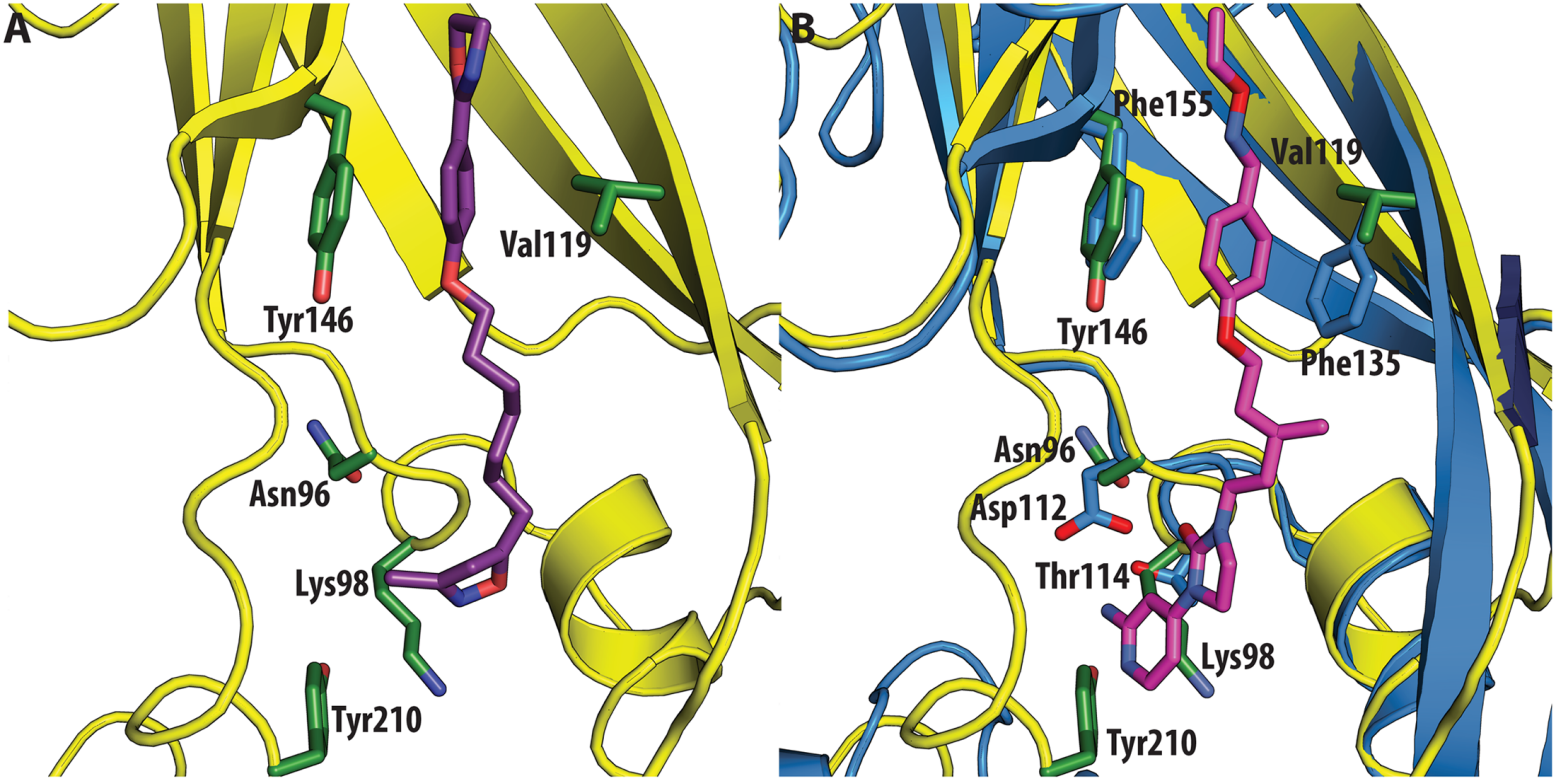
(**A**) VP1 pocket of CVA9. Inhibitor (violet) and residues side chains (green) are shown in sticks (PDBid:1D4M). (**B**) Superposition of CVA9 (yellow) on EV71 (blue) in complex with NLD (PDBid:4CEY). Ligand (magenta) and side chain residues (green for CVA9, blue for EV71) are shown as sticks. The residues shown in EV71 are: Asp112, Thr114, Phe135, Phe155. Residues shown in CVA9 are: Arg96, Lys98, Val119, Tyr146 and Tyr210.

In contrast, very low activity of the three capsid binders is observed against Coxsackievirus B3 (CVB3). This is likely due to the presence of Arg95 in CBV3 that would collide with the 2-amino-pyridine moiety of NLD, the 2-amide-pyridine moiety of ALD or the pyridine moiety of GPP3 (Fig 6). Residues Arg101 and Glu105, which are within a radius of 5Å, would prevent the movement of Arg95 (Fig 6), whereas Thr93, which is present in the pocket instead of Asp112 in the case of EV71 and CVA16 prevents the formation of hydrogen bonds with the amino group of NLD and amide group of ALD. Similarly, echovirus 11 has Tyr146 and Val119 at the bottom of the pocket (Fig 7), which only permits weaker hydrophobic interactions with the phenoxy moiety of the capsid binders; whilst Tyr210 constrains the size of the pocket factor that can be accommodated and, as a consequence, prevents binding of the capsid binders. Moreover, this residue is also involved in a stacking interaction with Arg98 and Arg104 (Fig 7): binding of the capsid binders would require the displacement of Tyr210, and disruption of the stacking interactions would incur a significant energetic penalty. Finally, the replacement of Asp112 by Ser96 prevents hydrogen-bonding with the amino group of NLD and the amide group of ALD. Similar structural features that interfere with the interaction with capsid binders are observed in Coxsackie A9 (CVA9). Tyr146 and Val119 at the bottom of the pocket reduce the hydrophobic interaction with the inhibitors, whilst the close contact of Tyr210 and Lys98 with the pyridine moiety of the inhibitors would hinder binding (Fig 8).

## Discussion

One of the most promising strategies to prevent infection with enteroviruses is to replace the hydrophobic-pocket-factors with more robust, high-affinity pocket binders[5,8]. We investigated the interaction of GPP3 with CVA16 by determining the crystal structure of it in complex with the whole virus particle and identified the key residues involved in the interactions with the capsid binder including Phe135 and Tyr155 which make a hydrophobic sandwich with the phenoxy moiety as observed for EV71[8]. The QMPLD[12] method, with guidance from the observed crystal structures, provided reliable docking results and identified the residues in the CVA16 pocket interacting with NLD and ALD.

**Fig 9:**
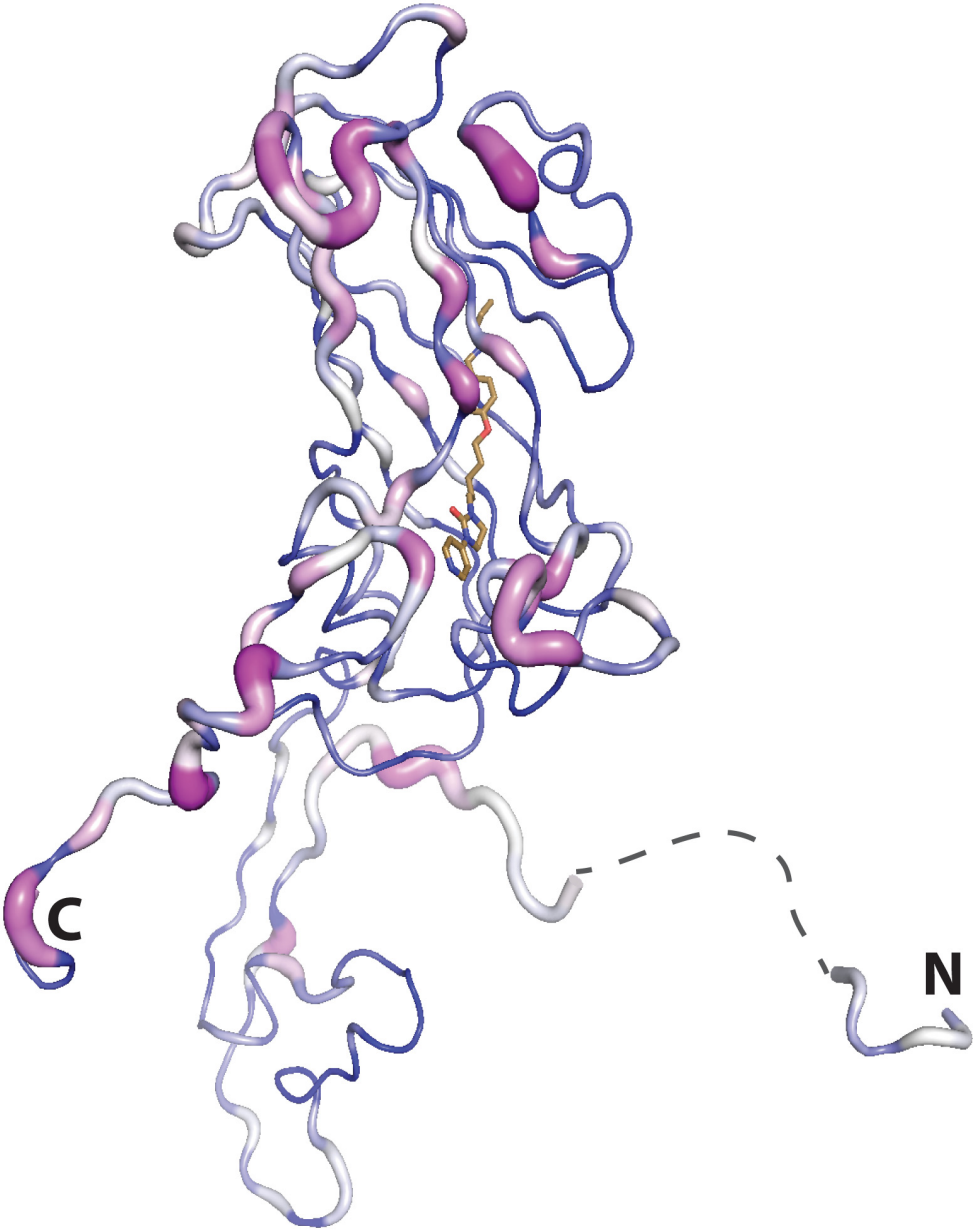
Residue conservation mapped onto the VP1 subunit of CVA16 (residues 2:8 and 19:297 the disordered portion spanning these residues is shown by a dashed line) using Consurf (violet = invariant, white = conserved, blue = variable), based on the alignment of all the virus structures used in the inhibitor assays. The N and C terimini are marked.

We supported these structural results with virus-cell-based assays using the respective inhibitors. The EC_50_ measurements performed with these inhibitors against CVA16, showed for NLD an EC_50_ of 0.12 nM. Additionally, we tested the activity of these inhibitors against a panel of enteroviruses (Table 2). Finally, structural comparisons (Fig 9) were used in a meta-analysis to correlate structural features with differences in antiviral activity. These results reinforce the potential of NLD as a candidate for a HFMD drug. We also rationalized the results for other viruses: low activity generally accompanies replacement of a Phe with Leu which fails to make the proper hydrophobic sandwich with the phenoxy moiety present in all inhibitors or the presence of side chains cluttering the entrance of the pocket interfering with the binding of the inhibitors. Good anti-poliovirus activity was observed for all the three capsid binders with highest EC_50_ for PV1 which has a Leu residue at the bottom of the pocket, that reduces the energy of binding, compared to PV2 and PV3, which have a Phe. In conclusion, the hydrophobic pocket below the canyon in VP1 still proves an interesting target for the development of novel inhibitors that target the early stage of enterovirus infection.

## Materials and Methods

### Virus production, purification and crystallization

CVA16 (genotype B) was isolated from Zhejiang Provence, China. The virus was grown in Vero cells (from the Shanghai Cell Bank of the Chinese Academy of Sciences) in Dulbecco’s modified Eagle’s medium (DMEM; Sigma) supplemented with 0.5% fetal bovine serum (FBS) (Gibco) until 90% of cells exhibited a cytopathic effect (CPE). Both cells and virus containing supernatant were collected, frozen and thawed three times, centrifuged to remove cell debris and ultra-filtered. The virus supernatant was concentrated and subjected to sucrose density gradient ultracentrifugation. CVA16 was inactivated by formaldehyde and purified as described previously[6]. Diamond-shaped crystals of CVA16 mature virions (at a concentration of 2 mg/ml in PBS buffer) with a maximum size of 0.1 × 0.1 × 0.08 mm^3^ grew in 3.2 M sodium chloride, 0.1 M sodium acetate trihydrate pH 7.0 (Screen SaltRx 1, Hampton Research, condition 12) within 2 weeks. GPP3 was dissolved in 100% DMSO at a concentration of 19 mg/ml, stock solution was mixed with the above mother liquor in a ratio of 1:2 and further diluted in water to give a solution containing 3.8 mg/ml ligand. About 0.5 μl of this solution was added to the 0.2-μl crystallization drops 1 week before data collection.

#### Structure determination

Data were collected *in situ[9]* on beamline I03 at Diamond light source. Diffraction images of 0.05° or 0.1° rotation were recorded on a Pilatus 6M detector using an unattenuated beam of 0.08 × 0.02 mm^2^ at I03, with exposure times of 0.1 s per image. Owing to radiation damage in the microcrystals, data collection was limited to 3–10 frames per crystal. Data processing was performed with HKL2000[16]. <I/σI> was calculated with ioversigma.py (http://strucbio.biologie.uni-konstanz.de/ccp4wiki/index.php/Calculate_average_I/sigma_from_.sca_file) and intensities converted to structure-factor amplitudes with TRUNCATE[17]. All crystals belonged to space group *P*4_1_2_1_2 with one particle in the asymmetric unit. Despite the low completeness of the data set the high non crystallographic redundancy allows to overcome efficiently the lack of experimental diffraction data. The data collected were isomorphous with those for the mature CVA16 virus in complex with sphingosine, therefore after removing the sphingosine that model was subjected to positional and B-factor refinement with NCS constraints in CNS.1.3[18]. NCS operators were updated by rigid-body refinement of individual protomers in PHENIX[19] and recalculated NCS matrices used as constraints in CNS.1.3[18], performing simulated annealing at 500K, positional and B-factor refinement. Water molecules were modeled into the 3.5σ peaks of an Fo − Fc map. Density modification was performed with CNS.1.3[18] and Coot[20]. Ligand coordinates were generated with PRODRG[21], and restraint dictionaries generated by Grade (http://grade.globalphasing.org/), PRODRG[21] and XPLO2D[22]. Model building was performed with Coot[20]. The Fo − Fc map was calculated removing the atoms corresponding to the pocket factor from the model before the phase calculation with CNS[18]. The electron density map was averaged according to 60-fold non crystallographic symmetry in Coot[20]. The model was validated with MolProbity[23]. 93.35% of the residues were in favored regions of the Ramachandran plot, and 0.25% were outliers. Figures were prepared with PyMOL (http://www.pymol.org/). Structures superposition was performed by SSM[24]. Structure sequence alignments was performed with Promals[25]. Mapping of residue conservation on the structure was performed by Consurf[26].

### Virus-cell-based assays

BGM, HeLa H1 (a subclone of HeLa cells highly sensitive to virus-induced cell death by CVA16), HeLa Rh (a subclone of HeLa cells highly sensitive to virus-induced cell death by rhinoviruses) and RD cells, subcultured in cell growth medium [MEM Rega3 (Cat. N°19993013; Invitrogen) or MEM (Cat. N°21090; Invitrogen) supplemented with 10% FCS (Integro), 5ml 200mM L-glutamine (25030024) and 5ml 7.5% sodium bicarbonate (25080060) at a ratio of 1:5 (BGM) or 1:10 (HeLa H1, HeLa Rh and RD) and grown for 7 (BGM) or 3–4 (HeLa H1, HeLa Rh and RD) days in 150cm^2^ tissue culture flasks (Techno Plastic Products), were harvested and a cell suspension was prepared with a cell density of 25,000 cells/50μl in assay medium (MEM Rega3, 2% FCS, 5ml L-glutamine and 5ml sodium bicarbonate) of which 50μl was seeded per well at the end of the assay setup. Compound dilutions were prepared in assay medium added to empty wells (96-well microtiter plates, Falcon, BD). Subsequently, 50μl of a 4x virus dilution in assay medium (assay medium supplemented with 15ml MgCl 1M (Sigma, M1028) in case of HRV) was added followed by addition of 50μl of cell suspension. The assay plates were returned to the incubator for 3–4 days, (35°C for HRV) at which time maximal cytopathic effect is observed. For the evaluation of cytostatic/cytotoxic effects and for the evaluation of the antiviral effect, the assay medium was aspirated, replaced with 75μl of a 5% MTS (Promega) solution in phenol red-free medium and incubated for 1.5 hours (37°C, 5% CO2, 95–99% relative humidity). Absorbance was measured at a wavelength of 498nm (Safire^2^, Tecan) and optical densities (OD values) were converted to percentage of untreated controls. The EC_50_ (50% effective concentration) and CC_50_ ± SD (50% cellular cytotoxicity) were, whenever possible, calculated respectively as the median of all the EC_50_ or CC_50_ values derived from at least 3 individual dose-response curves. Following quantitative data collection, each well, in which >50% cell survival was measured, was checked by microscope for minute signs of virus-induced cytopathic effects or alterations to the cell or monolayer morphology. A compound was only considered a selective inhibitor of virus replication when at least one treated, infected condition resembled the untreated, uninfected cell control.

### Molecular docking and binding-energy calculation

Small-molecule coordinates were generated by PRODRG24 and energy minimized with Ligprep in the Schrödinger suite at pH 7.0 with the OPLS_2005 force field[27]. The standard conversion procedure with full hydrogen optimization was applied with the Protein Preparation workflow. The VP1 binding pocket in the crystal structure in complex with the GPP3 ligand was taken as the receptor structure. These processed coordinates were used for the subsequent grid generation and ligand-docking procedures. The Glide Grid[28,29] (Schrödinger suite) was built using an inner box (the centroid of the GPP3 molecule) of 8 × 8 × 8 Å^3^ and an outer box (within which all the ligand atoms must be contained) that extended 30Å in each direction from the inner one. Default values were used for all other parameters. The hydrogen bond between the imidazole moiety of the GPP3 molecule and the carbonyl group of VP1 Ile113 and hydrophobic constraints corresponding to the region identified as a hydrophobic trap were used as positional constraints. For docking, the QMPLD[12] protocol (Schrödinger suite, http://www.schrodinger.com/) was used. This ligand docking protocol aims to improve the partial charges on the ligand atoms in the docking by replacing them with charges derived from quantum mechanical calculations. In this hypothesis, the use of mixed quantum mechanical/molecular mechanics (QM/MM) model for the ligand charges are employed in docking calculations rather than usual fixed charges assigned by force field such as OPLS. These force fields use charges derived by empirical methods that don’t account role of polarization of ligand in specific environments. Employment of QM/MM techniques enables the charge calculations for the ligand to be performed in the protein environment, thus incorporating polarization effects in a natural and accurate fashion[30]. In this way, the polarization of the charge on the ligand by receptor is accounted for, resulting in an improved docking pose. The most reliable binding pose for each small molecule was selected on the basis of calculated van der Waals and electrostatic interactions. RMSD values were calculated with VMD[31].
